# Cancer-associated thrombotic microangiopathy

**DOI:** 10.3332/ecancer.2016.649

**Published:** 2016-06-28

**Authors:** K Govind Babu, Gita R Bhat

**Affiliations:** Department of Medical Oncology, Kidwai Memorial Institute of Oncology, Dr MH Marigowda road, Hombegowdanagar, Bangalore-560029, India

**Keywords:** thrombotic microangiopathy, haemolytic uraemic syndrome, thrombotic thrombocytopenic purpura, gemcitabine, mitomycin-C, haematopoietic stem cell transplant, immunotoxins, anti-VEGF, plasmapheresis

## Abstract

Cancer-associated thrombotic microangiopathy refers to a group of disorders characterised by microvascular thrombosis, thrombocytopenia, and ischaemic end-organ damage. Haemolytic uraemic syndrome and thrombotic thrombocytopenic purpura are the two major subtypes. It can be a manifestation of the malignancy itself or a complication of its therapy. The addition of several new drugs to the therapeutic armamentarium of cancer has brought to light several novel causative factors of this hitherto uncommon complication. This review covers the aetiology, pathogenesis, clinical manifestations, complications, and the management of cancer-associated thrombotic microangiopathy. Careful review of the patient’s medical records coupled with the correlation of clinical findings and laboratory reports can help clinch the diagnosis and institute appropriate treatment on time.

## Introduction

Thrombotic microangiopathies (TMA) are a group of disorders characterised by disseminated occlusive microvascular thrombosis, thrombocytopenia, and ischaemic end-organ damage, most commonly in kidneys and brain. Haemolytic uraemic syndrome (HUS) and thrombotic thrombocytopenic purpura (TTP) are the two main subtypes [1].

TTP is described by a pentad of clinical findings including thrombocytopenia, microangiopathic haemolytic anaemia (MAHA), neurologic deficits, such as seizures, hemiplegia and visual disturbances, renal failure, and fever. HUS is defined as a triad of MAHA with varying degrees of thrombocytopenia and renal failure [1, 2]. Fragmentation of erythrocytes as they pass through clogged arterioles gives rise to Coombs-negative haemolytic anaemia with an elevated schistocyte count. Renal insufficiency invariably occurs in HUS but is milder in TTP, where neurological symptoms often predominate [1]. The pathological features include intra-renal or systemic microvascular thrombi with endothelial swelling and microvascular obstruction [3].

von Willebrand factor (vWF) is initially secreted from Wiebel–Palade bodies as multimers tethered to the endothelial cell surface. Here, they provide glycoprotein Ib receptor sites for platelet adhesion and thrombus formation. ADAMTS13, also called von Willebrand factor-cleaving protease (vWF-cp) regulates thrombus formation by cleaving the multimeric vWF [4, 5]. Severe deficiency of ADAMTS13 (<5% of normal) has been proposed as the key pathogenetic factor for idiopathic TTP [6]. Studies have shown that cancer-associated TMA is not associated with the severe deficiency of ADAMTS13 [7].

## History of cancer-associated thrombotic microangiopathy

The term ‘microangiopathic haemolytic anaemia’ was coined by Brain *et al* in 1962. They observed that ‘widespread intra-capillary and intra-arteriolar eosinophilic granular or amorphous thrombi’ could reduce the lumen of the blood vessels to ‘pin-point size’, leading to mechanical red cell destruction by intra-luminal shearing. They described the appearance of fragmented red cells as ‘burr, triangular, and helmet red cells’ [8]. In their series of 25 patients, the various conditions that caused MAHA were TTP, malignant hypertension, acute renal disease, and metastatic carcinomas. In 1979, Antman *et al*. in the review on MAHA and cancer proposed that the haemolysis and thrombocytopenia were primarily caused by mechanical obstruction of the vascular lumen by tumour cell emboli. They concluded that a variety of systemic malignancies could cause clinicopathological features of TMA without coagulation abnormalities like disseminated intravascular coagulation [9].

## Aetiopathogenesis

In patients with cancer, TMA have been reported as follows:

(1) A manifestation of cancer itself:

Most cases of cancer-associated TMA have been reported in patients with mucin-producing adenocarcinoma and in those with disseminated malignancies. It has also been described in cases with isolated invasion of the bone marrow by the tumour [10]. The incidence of TMA in this population is said to range from 0.25 to 0.45 persons per million [11]. A prospective study by Lohrmann *et al*. determined that 5.7% of patients with metastatic carcinoma have MAHA [10]. In an extensive review by Lechner *et al*., adenocarcinoma was the most common histopathological subtype in patients with cancer-associated TMA. Gastric carcinoma was the most common (26.2%), followed by breast (21.4%), prostate (13.7%), and lung cancer (9.5%) [11]. It has also been reported in pancreatic adenocarcinoma, lymphoma as well as other malignancies [12]. The malignancies associated with MAHA are listed in Table 1 [8, 9, 11, 13–21].

It has been postulated that, along with abnormal angiogenesis in the marrow, aggressive growth of tumours and secondary myelofibrosis may injure the endothelial cell lining of the marrow vasculature by direct invasion. This could result in release of ultra large VWF multimers (ULVWF). In addition, a possible decrease in availability of VWF-cleaving protease (ADAMTS13), possibly through formation of autoantibodies against ADAMTS13 may contribute to the development of TMA. Fragmentation of red blood cells due to direct contact with intraluminal fibrin thrombi or tumour emboli within blood vessels may lead to MAHA [22]. The effect of mucin on endothelial dysfunction has also been proposed as a mechanism for the development of TMA [23].

(2) As a complication of chemotherapy:

Chemotherapy may cause TMA by two mechanisms, namely an acute immune-mediated reaction or dose-dependent toxicity. Acute and presumed immune-mediated TMA has been reported with oxaliplatin [24]. Oxaliplatin-dependent, platelet-reactive antibodies have been documented in patients with acute oxaliplatin-induced thrombocytopenia [25].

The incidence of gemcitabine induced HUS ranges from 0.015% to 0.31%. The median duration of therapy is 5.8 months, with most of the patients developing HUS within one to two months of the last infusion. It develops after a median cumulative dose of 20 g/m^2^, with a broad range from 2.5 g/m^2^ to 48 g/m^2^. Mortality may be as high as 60% [26–30]. Although gemcitabine-induced TMA is typically dose-dependent, there are case reports of acute TMA following the first or second infusion [31]. It is said to occur due to uncontrolled proximal alternative pathway complement activation. This leads to an increased terminal membrane attack complex giving rise to endothelial cell activation. There is activation of monocytes, neutrophils, and platelet activation and aggregation. Gemcitabine may directly damage endothelial cells, resulting in platelet aggregation and intravascular haemolysis [32].

The incidence of mitomycin-C-induced HUS ranges from 2% to 15%. The risk increases significantly when the cumulative dose exceeds 30 mg/m^2^ to 40 mg/m^2^ and after one year of treatment. The prognosis is poor with 75% mortality related to renal failure. Direct endothelial cell injury likely plays a central role. Following endothelial injury and exposure of the subendothelium, activation of platelets and the clotting cascade may occur [27, 28, 30, 33].

The other chemotherapeutic agents that have been implicated in the aetiology of HUS include 5-fluorouracil, bleomycin, cisplatin, cytosine arabinoside, daunomycin, deoxycoformycin, estramustine, and methyl-CCNU [34, 35].

(3) In the setting of bone marrow transplantation:

Chronic kidney disease (CKD) is an important long-term complication of haematopoietic stem cell transplant (HCT), particularly allogeneic in which it develops in 15% to 20% of survivors [36]. Most of the cases of CKD after HCT are thought to be related to a low-grade TMA. However, the incidence in patients undergoing bone marrow transplantation is uncertain, given the difficulty in establishing a diagnosis [37]. It is characterised by slowly rising serum creatinine, hypertension, and disproportionate anaemia. Some cases might have an acute, fulminant presentation with associated renal dysfunction and neurologic manifestations that may represent endothelial damage driven by donor–host interactions [38]. Careful review of previous reports reveals intermittent or persistent elevation of lactate dehydrogenase, low serum haptoglobin, thrombocytopenia, anaemia, and sometimes schistocytosis [39].

Exposure of the kidneys to radiation as a part of the pre-HCT conditioning damages the renal microvasculature. This is the primary cause of renal damage. Concurrent chemotherapy and genetic factors (angiotensin-converting enzyme genotype) play a modulatory role along with post-HCT factors such as use of calcineurin inhibitors (especially sirolimus), the presence of graft versus host disease, the presence of procoagulant state and infection [39–42]. Sirolimus inhibits VEGF resulting in glomerular podocyte injury [43, 44]. Damage to endothelial cells along with cell swelling and fibrin deposition causes microvascular occlusion and results in haemolysis. Glomerular ischaemia gives rise to hypertension and over time leads to fibrosis and renal failure [45].

(4) In those receiving antibodies and immunotoxins:

HUS has been reported in adults and children treated with immunotoxins and is generally reversible [46]. The causative agents include anti-CD 22 immunotoxins CAT-3888, formerly called BL22 [47–49] and moxetumomab pasudotox [50], combotox which is a 2 deglycosylated ricin A chain immunotoxins directed against CD 19 and CD 22 and DAB_486_IL-2, a recombinant immunotoxin that targets IL-2 [51].

The mechanism of immunotoxin-induced TMA is not well understood. In ricin-induced HUS as demonstrated in animal models, upregulation of pro-inflammatory cytokines MCP-1, TNF-α, IL-1β, and IL-6 promotes an inflammatory environment. This leads to infiltration of the glomeruli with macrophages that stimulate secretion of vWF. This leads to the development of glomerular thrombosis, renal failure, haemolysis, and thrombocytopenia [52, 53]. In case of anti-CD 22 immunotoxins cross reactivity to a different target or non-specific uptake into cells by pinocytosis may play a role [54].

HUS is a dose-limiting toxicity of apolizumab, a humanised monoclonal antibody against an antigen on the β-chain of HLA-DR called 1D10. Alemtuzumab, an anti-CD 52 monoclonal antibody has also been known to induce HUS. It is reversible [55]. Release of cytokines, TNF-α and IL-6 may be causative.

## Anti-VEGF therapy

Several anti-VEGF agents, such as bevacizumab [44], sunitinib [56–58], and aflibercept [59], have been associated with TMA, suggesting a potential classwide effect. Eremina *et al*. have described six biopsy documented cases of TMA-resembling HUS following administration of bevacizumab. These patients developed proteinuria or raised serum creatinine following the initiation of bevacizumab, ultimately leading to renal biopsy. Following discontinuation of therapy, improvement in renal function was noted, suggesting at least partial reversibility.

It has been proposed that inhibition of VEGF in the glomerular microvasculature prevents the formation and maintenance of a healthy, fenestrated endothelium. Without active VEGF signalling, the endothelium is compromised. This leads to loss of integrity of the glomerular filtration barrier [44].

## Imatinib

Al Aly *et al*. have published a case report on a 22-year-old lady with hypereosinophilic syndrome who developed TMA while on Imatinib. ADAMTS13 activity was below the normal range. There was marked elevation of ADAMTS13 inhibitor levels. She was treated with plasma exchange and haemodialysis, leading to haematological recovery but persistent renal impairment [60].

## Diagnosing thrombotic microangiopathy

The diagnosis of TMA is based on recognising microvascular thrombosis, red blood cell destruction, and platelet consumption. At present, there are no guidelines to establish the diagnosis.

Blake–Haskins *et al*. have proposed a detailed grading of TMA. Grade 1 refers to evidence of erythrocyte destruction, without clinical consequences. It has been described as the appearance of schistocytes (≥ 5/HPF) in a patient with no schistocytes at baseline or an increase (≥ 5/HPF) in the frequency of schistocytes over the baseline. Grade 2 includes laboratory findings, such as schistocytosis, drop in haemoglobin by 1 g/dL, and mild renal insufficiency, without clinical consequences. Grade 3 refers to laboratory findings along with clinical consequences. The patient has schistocytosis and drop in haemoglobin by 1 g/dL and either grade 4 thrombocytopenia or moderate renal insufficiency. Grade 4 TMA includes to the presence of life-threatening consequences such as clinically significant haemorrhage requiring intervention, thrombosis, and/or embolism or renal insufficiency requiring intervention. Grade 5 refers to death due to TMA [61].

The clinical features of fulminant HUS/TTP include the classic pentad of MAHA, thrombocytopenia, fever, rapidly progressive renal failure, and neurological deficits [1]. Some may have a subacute presentation with mild thrombocytopenia and gradual deterioration of renal function [1]. Renal dysfunction is seen in almost all the cases and is manifested by elevations in serum creatinine and/or worsening haematuria. Evidence of haemolysis such as fall in haemoglobin, low levels of haptoglobin; elevation of LDH and reticulocyte count and indirect hyperbilirubinaemia is seen frequently. Identification of schistocytes in by peripheral smear may not be documented in all cases.

In the series by Humphreys *et al*., new or exacerbated hypertension was a prominent feature in gemcitabine-induced HUS. It is important to note that this feature preceded the diagnosis of HUS in all cases. The severity of hypertension correlates with poor outcome [39]. Hypertension in TMA is due to glomerular ischaemia caused by microvascular capillary obstruction [45].

The differences between MAHA caused by systemic malignancy and TTP associated with severe ADAMTS13 deficiency, as per the Oklahoma TTP-HUS registry are as follows [13, 14, 62]:

- Patients with cancer-associated MAHA present at an older ager (mean age: 56 years) versus those with TTP associated with severe ADAMTS13 deficiency (mean age: 40 years).
- We must look for the evidence of active malignancy in those with a history of cancer. Even if the cancer appears to be in remission, evidence of recurrence, such as bone marrow biopsy is necessary.
- Progressive weakness, weight loss, and pain are common in patients with cancer-associated MAHA. They have a longer duration of symptoms (median duration: 21 days) versus those with TTP associated with ADAMTS13 deficiency (median duration: 8 days).
- Pulmonary involvement characterised by dyspnoea, cough, and abnormal chest X-ray is a feature of cancer-associated MAHA.
- Leucoerythroblastic reaction and extreme and extreme elevation of LDH are seen in those with cancer-associated MAHA.
- The median value of ADAMTS13 activity is 50% in those with cancer-associated MAHA.
- MAHA associated with systemic malignancies fails to respond to plasma exchange. On the other hand, patients with TTP due to ADAMTS13 deficiency respond promptly with rapid reversal of neurologic symptoms, reduction of LDH levels over 1 to 2 days and rise in platelet counts over 3 to 4 days.

## Is renal biopsy always required?

Renal biopsy is very rarely required, unless the presentation is atypical. The clinical and laboratory features often point to a diagnosis of HUS/TTP. Biopsy findings do not significantly alter management. Biopsy is an invasive procedure and carries increased risk in these patients with thrombocytopenia and other comorbidities. The typical histopathological features are mesangiolysis, basement membrane duplication, glomerular endothelial cell swelling, and tubular injury with interstitial fibrosis [63].

## Role of bone marrow biopsy

Patients with history of cancer, including those who appear to be in complete remission need an extensive workup, including bone marrow biopsy to look for the evidence of malignancy [13]. Marrow infiltration by metastatic carcinoma or lymphoma may lead to thrombocytopenia and MAHA [14–16].

## Complications

Acute respiratory distress syndrome may develop in patients with a fulminant course. It is associated with a high mortality rate because treatment is even more difficult in a patient with underlying disease [1]. Seizures have been reported in those with the involvement of the central nervous system (CNS). Pancreatic insufficiency may occur, which is manifested as diabetes mellitus, which usually is transient. The muscle, liver, and heart (cardiomyopathy and myocarditis) also can be affected [2]. CKD with hypertension is one of the long-term outcomes [39]. End-stage renal disease (ESRD) may develop.

## Differential diagnosis

HUS should be differentiated from isolated renal insufficiency in the presence of myelotoxicity. In the latter scenario, patients usually do not have laboratory evidence of haemolysis with microangiopathy (schistocytes, raised reticulocyte count, indirect hyperbilirubinaemia, elevated lactate dehydrogenase or fibrin split products). Anaemia and thrombocytopenia associated with myelosuppression tend to be more severe. Mild renal insufficiency unrelated to HUS should resolve quickly or return to baseline on rehydration or treatment of the underlying pre-renal state. In renal failure unrelated to HUS, renal biopsy, if performed, will not show the classic microvascular damage with arterioles and small arteries occluded by eosinophilic hyaline thrombi containing fibrin and platelet aggregates [64]. In patients with malignancy, the other causes of thrombocytopenia and haemolysis include disseminated intravascular coagulation and sepsis.

## Treatment and outcome

Is there a scope for early detection and prevention of TMA?

In case of gemcitabine induced HUS, early diagnosis may be aided by weekly visits to the chemotherapy day care by the patient might give a chance to detect new or exacerbated hypertension as it develops [65]. Renal shielding during total body irradiation in the setting of allogeneic HCT is said to be protective [66].

As of today, there is no definitive approach for cancer-associated TMA. Treatments comprise of the following main categories:

1. When a given therapy is implicated as causative, it should be discontinued. Reintroducing the drug at a lower dose may be a strategy to avoid recurrent TMA, while allowing for continued treatment. Retreatment has been successfully carried out with CAT-3888, DAB_486_IL-2, and bevacizumab. However, it may be difficult to choose an appropriate dose for reintroduction and the risks and benefits involved need to be carefully assessed [44, 49, 67].
2. Immunocomplex removal: This includes plasmapheresis, immunoadsorption, haemodialysis, or exchange transfusion. Although the efficacies of plasma exchange and immunoadsorption chromatography are arguable, either or both should be initiated promptly at diagnosis in all patients. These may need to be continued for months [11].
3. Immunosuppressive therapies: The value of corticosteroids is uncertain [1].
4. Miscellaneous: Blood pressure should be controlled.
5. Eculizumab: It is a recombinant humanised monoclonal antibody that binds to complement C5 protein, inhibiting its cleavage and thus preventing the generation of the terminal complement attack complex C5b-9. The common adverse events are headache, anaemia, and diarrhoea. Neisseria meningitides vaccination is indicated at least two weeks prior to treatment. It was initially approved for the treatment of paroxysmal nocturnal haemoglobinuria and has also been used in atypical HUS.

Al Ustwani *et al*. have reported the use of eculizumab in four patients with gemcitabine-induced HUS. In their series, patients were initiated upon eculizumab as the patients did not show improvement of renal function or haemolysis even after administration of corticosteroids stopping gemcitabine for three to five weeks. It resulted in the resolution of microangiopathic haemolysis and thrombocytopenia in all four patients. All patients had significant improvement in renal function but did not return to baseline. None of the patients had serious adverse events [32].

## Conclusion

The diagnosis of cancer-associated TMA needs a high index of suspicion. Careful review of diagnosis of cancer, treatment history and interpretation of subtle clinical signs will aid in clinching the diagnosis. Patients with smaller tumour burden, or those in whom anticancer therapy is effective, and in cases where vigilance has led to early diagnosis tend to do better. It has also been suggested that prognosis is better when TMA occurs as a manifestation of the underlying cancer than as a complication of therapy.

## Abbreviations used in this article

ADAMTS13: A disintegrin-like and metalloprotease with thrombospondin type-1 repeats

CKD: Chronic kidney disease

HUS: Haemolytic uraemic syndrome

MAHA: Microangiopathic haemolytic anaemia

TMA: Thrombotic microangiopathy

TTP: Thrombotic thrombocytopenic purpura

ULVWF multimers: Ultra large von Willebrand factor multimers

vWF: von Willebrand factor

vWF-cp: von Willebrand factor-cleaving protease

## Figures and Tables

**Table 1.** Systemic malignancies associated with microangiopathic haemolytic anaemia [8, 9, 11, 13–21].

Solid tumours:	
Gastrointestinal malignancies	Gastric cancer
	Colon cancer
	Carcinoma of anal canal (squamous cell)
	Metastatic appendiceal carcinoma
Breast cancer	
Lung cancer	Adenocarcinoma
	Squamous cell carcinoma
	Small cell lung cancer
Genitourinary malignancies	Prostate cancer
	Ovarian cancer
	Renal cell carcinoma
	Seminal vesicle tumour
Hepatobiliary cancers	Hepatocellular carcinoma
	Pancreatic cancer
	Cholangiocarcinoma
Endocrine	Multiple endocrine neoplasia type 1
	Pheochromocytoma
	Neuroendocrine tumour
	Prolactin producing pituitary adenoma
Others	Kaposi sarcoma
	Carcinoma of unknown origin
**Hematologic malignancies:**	
Non-Hodgkin lymphoma	
Acute lymphoblastic leukaemia	
Myelodysplastic syndrome	
Hodgkin lymphoma	
Multiple myeloma	
